# 2-(4-Fluoro­phen­yl)-5,6-methyl­enedi­oxy-3-phenyl­sulfinyl-1-benzofuran monohydrate

**DOI:** 10.1107/S160053681105553X

**Published:** 2012-01-14

**Authors:** Pil Ja Seo, Hong Dae Choi, Byeng Wha Son, Uk Lee

**Affiliations:** a Department of Chemistry, Dongeui University, San 24 Kaya-dong Busanjin-gu, Busan 614-714, Republic of Korea; b Department of Chemistry, Pukyong National University, 599-1 Daeyeon 3-dong, Nam-gu, Busan 608-737, Republic of Korea

## Abstract

In the title compound, C_21_H_13_FO_4_S·H_2_O, the dihedral angles between the mean plane of the benzofuran fragment (r.m.s. deviation = 0.005 Å) and the pendant 4-fluoro­phenyl and phenyl rings are 6.24 (7) and 83.39 (6)°, respectively. In the crystal, mol­ecules are linked by O—H⋯O and C—H⋯O hydrogen bonds.

## Related literature

For the pharmacological activity of benzofuran compounds, see: Aslam *et al.*(2009 ▶); Galal *et al.* (2009 ▶); Khan *et al.* (2005 ▶). For natural products with benzofuran rings, see: Akgul & Anil (2003 ▶); Soekamto *et al.* (2003 ▶). For the crystal structure of related compound, see: Choi *et al.* (2009 ▶).
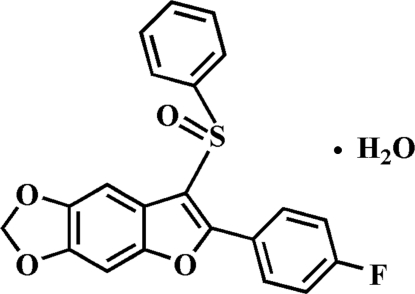


## Experimental

### 

#### Crystal data

C_21_H_13_FO_4_S·H_2_O
*M* *_r_* = 398.39
Monoclinic, 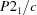

*a* = 8.2485 (2) Å
*b* = 33.5624 (9) Å
*c* = 6.1854 (2) Å
β = 93.001 (2)°
*V* = 1710.01 (8) Å^3^
*Z* = 4
Mo *K*α radiation
μ = 0.23 mm^−1^
*T* = 173 K
0.39 × 0.16 × 0.11 mm

#### Data collection

Bruker SMART APEXII CCD diffractometer
Absorption correction: multi-scan (*SADABS*; Bruker, 2009 ▶) *T*_min_ = 0.915, *T*_max_ = 0.975
16270 measured reflections
3947 independent reflections
3235 reflections with *I* > 2σ(*I*)
*R*_int_ = 0.036

#### Refinement

*R*[*F* ^2^ > 2σ(*F* ^2^)] = 0.048
*wR*(*F* ^2^) = 0.120
*S* = 1.02
3947 reflections
261 parameters
H atoms treated by a mixture of independent and constrained refinement
Δρ_max_ = 0.94 e Å^−3^
Δρ_min_ = −0.61 e Å^−3^

### 

Data collection: *APEX2* (Bruker, 2009 ▶); cell refinement: *SAINT* (Bruker, 2009 ▶); data reduction: *SAINT*; program(s) used to solve structure: *SHELXS97* (Sheldrick, 2008 ▶); program(s) used to refine structure: *SHELXL97* (Sheldrick, 2008 ▶); molecular graphics: *ORTEP-3* (Farrugia, 1997 ▶) and *DIAMOND* (Brandenburg, 1998 ▶); software used to prepare material for publication: *SHELXL97*.

## Supplementary Material

Crystal structure: contains datablock(s) I, global. DOI: 10.1107/S160053681105553X/lr2043sup1.cif

Structure factors: contains datablock(s) I. DOI: 10.1107/S160053681105553X/lr2043Isup2.hkl

Additional supplementary materials:  crystallographic information; 3D view; checkCIF report

## Figures and Tables

**Table 1:** Hydrogen-bond geometry (Å, °)

*D*—H⋯*A*	*D*—H	H⋯*A*	*D*⋯*A*	*D*—H⋯*A*
C18—H18⋯O4^i^	0.95	2.46	3.375 (3)	162
C19—H19⋯O5w^i^	0.95	2.49	3.433 (3)	171
O5*W*—H5*WA*⋯O4^ii^	0.99 (4)	1.87 (4)	2.834 (2)	164 (3)
O5*W*—H5*WB*⋯O4^iii^	0.97 (4)	1.98 (4)	2.908 (2)	161 (3)

Symmetry codes: (i) 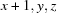; (ii) 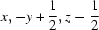; (iii) 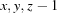.

**Fig. 1.**
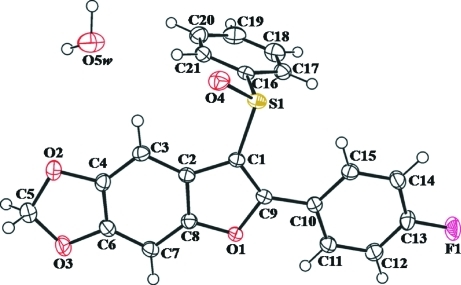
The molecular structure of the title compound. Displacement ellipsoids are drawn at the 50% probability level.

**Fig. 2.**
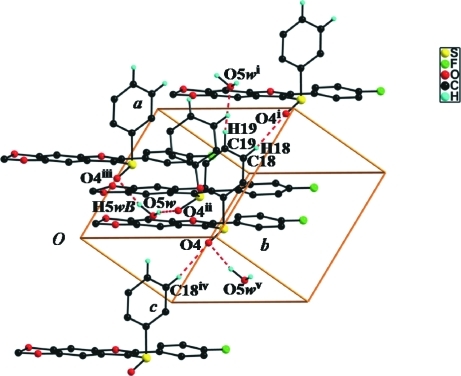
A view of crystal packing showing the O—H···O and C—H···O hydrogen bonds (dotted lines).H atoms non-participating in hydrogen-bonding were omitted for clarity. [Symmetry codes: (i) x + 1, y, z (ii) x, - y + 1/2, z - 1/2 (iii) x, y, z - 1 (iv) x - 1, y, z; (v) x, y, z + 1.]

## supplementary crystallographic information

### Comment

Recently, many compounds having a benzofuran ring have drawn much attention due to their valuable pharmacological properties such as antibacterial and antifungal, antitumor and antiviral, and antimicrobial activities (Aslam *et al.*, 2009, Galal *et al.*, 2009, Khan *et al.*, 2005). These benzofuran derivatives occur in a wide range of natural products (Akgul & Anil, 2003; Soekamto *et al.*, 2003). As a part of our ongoing studies of 5,6-(methylenedioxy)benzofuran derivatives containing 2-(4-bromophenyl) (Choi *et al.*, 2009) substituents, we report herein the crystal structure of the title compound.

The title compound crystallizes as a hydrate (Fig. 1). The benzofuran unit is essentially planar, with a mean deviation of 0.005 (1) Å from the least-squares plane defined by the nine constituent atoms. The dihedral angles between the mean plane of the benzofuran fragment and the pendant 4-fluorophenyl and phenyl rings are 6.24 (7) and 83.39 (6)°, respectively. The crystal packing (Fig. 2) is stabilized by intermolecular O—H···O and C—H···O hydrogen bonds (see Table 1).

### Experimental

77% 3-chloroperoxybenzoic acid (224 mg, 1.0 mmol) was added in small portions to a stirred solution of 2-(4-fluorophenyl)-5,6-methylenedioxy-3-phenylsulfanyl-1-benzofuran (291 mg, 0.8 mmol) in dichloromethane (30 ml) at 273 K. After being stirred at room temperature for 3 h, the mixture was washed with saturated sodium bicarbonate solution and the organic layer was separated, dried over magnesium sulfate, filtered and concentrated at reduced pressure. The residue was purified by column chromatography (hexane–ethyl acetate, 1:2 *v*/*v*) to afford the title compound as a colorless solid [yield 72%, m.p. 451–453 K; *R*_f_ = 0.79 (hexane–ethyl acetate, 1:2 *v*/*v*)]. Single crystals suitable for *X*-ray diffraction were prepared by slow evaporation of a solution of the title compound in ethyl acetate at room temperature.

### Refinement

The H atoms bonded to O5*w* were located a different Fourier map and refined freely. All other H atoms were positioned geometrically and refined using a riding model, with C—H = 0.93 Å fo the aryl and 0.97 Å for the methylene H atoms. *U*_iso_(H) =1.2*U*_eq_(C) for aryl and methylene H atoms.

### Crystal data

**Table:**

C_21_H_13_FO_4_S·H_2_O	*F*(000) = 824
*M**_r_* = 398.39	*D*_x_ = 1.547 Mg m^−^^3^
Monoclinic, *P*2_1_/*c*	Mo *K*α radiation, λ = 0.71073 Å
Hall symbol: -P 2ybc	Cell parameters from 4953 reflections
*a* = 8.2485 (2) Å	θ = 2.4–28.3°
*b* = 33.5624 (9) Å	µ = 0.23 mm^−^^1^
*c* = 6.1854 (2) Å	*T* = 173 K
β = 93.001 (2)°	Block, colourless
*V* = 1710.01 (8) Å^3^	0.39 × 0.16 × 0.11 mm
*Z* = 4	

### Data collection

**Table:**

Bruker SMART APEXII CCD diffractometer	3947 independent reflections
Radiation source: rotating anode	3235 reflections with *I* > 2σ(*I*)
graphite multilayer	*R*_int_ = 0.036
Detector resolution: 10.0 pixels mm^-1^	θ_max_ = 27.6°, θ_min_ = 1.2°
φ and ω scans	*h* = −10→9
Absorption correction: multi-scan (*SADABS*; Bruker, 2009)	*k* = −43→35
*T*_min_ = 0.915, *T*_max_ = 0.975	*l* = −8→7
16270 measured reflections	

### Refinement

**Table:**

Refinement on *F*^2^	Primary atom site location: structure-invariant direct methods
Least-squares matrix: full	Secondary atom site location: difference Fourier map
*R*[*F*^2^ > 2σ(*F*^2^)] = 0.048	Hydrogen site location: difference Fourier map
*wR*(*F*^2^) = 0.120	H atoms treated by a mixture of independent and constrained refinement
*S* = 1.02	*w* = 1/[σ^2^(*F*_o_^2^) + (0.0512*P*)^2^ + 1.6272*P*] where *P* = (*F*_o_^2^ + 2*F*_c_^2^)/3
3947 reflections	(Δ/σ)_max_ < 0.001
261 parameters	Δρ_max_ = 0.94 e Å^−^^3^
0 restraints	Δρ_min_ = −0.61 e Å^−^^3^

### Special details

**Table:**

Geometry. All e.s.d.'s (except the e.s.d. in the dihedral angle between two l.s. planes) are estimated using the full covariance matrix. The cell e.s.d.'s are taken into account individually in the estimation of e.s.d.'s in distances, angles and torsion angles; correlations between e.s.d.'s in cell parameters are only used when they are defined by crystal symmetry. An approximate (isotropic) treatment of cell e.s.d.'s is used for estimating e.s.d.'s involving l.s. planes.
Refinement. Refinement of *F*^2^ against ALL reflections. The weighted *R*-factor *wR* and goodness of fit *S* are based on *F*^2^, conventional *R*-factors *R* are based on *F*, with *F* set to zero for negative *F*^2^. The threshold expression of *F*^2^ > σ(*F*^2^) is used only for calculating *R*-factors(gt) *etc*. and is not relevant to the choice of reflections for refinement. *R*-factors based on *F*^2^ are statistically about twice as large as those based on *F*, and *R*- factors based on ALL data will be even larger.

### Fractional atomic coordinates and isotropic or equivalent isotropic displacement parameters (Å^2^)

**Table:**

	*x*	*y*	*z*	*U*_iso_*/*U*_eq_	
S1	0.50867 (6)	0.170330 (15)	0.88006 (8)	0.02163 (14)	
F1	0.92782 (18)	0.04232 (4)	1.5856 (2)	0.0415 (4)	
O1	0.49612 (16)	0.05550 (4)	0.7261 (2)	0.0205 (3)	
O2	0.14035 (19)	0.12607 (5)	0.0740 (3)	0.0318 (4)	
O3	0.1737 (2)	0.05788 (5)	0.0443 (3)	0.0330 (4)	
O4	0.36252 (17)	0.19343 (4)	0.8043 (3)	0.0283 (3)	
C1	0.4801 (2)	0.12171 (6)	0.7748 (3)	0.0181 (4)	
C2	0.3882 (2)	0.11212 (6)	0.5762 (3)	0.0191 (4)	
C3	0.2973 (2)	0.13442 (6)	0.4188 (3)	0.0210 (4)	
H3	0.2838	0.1625	0.4280	0.025*	
C4	0.2305 (2)	0.11216 (6)	0.2522 (3)	0.0222 (4)	
C5	0.0887 (3)	0.09168 (7)	−0.0456 (4)	0.0271 (5)	
H5A	0.1125	0.0949	−0.1998	0.033*	
H5B	−0.0298	0.0879	−0.0365	0.033*	
C6	0.2492 (2)	0.07098 (6)	0.2341 (3)	0.0230 (4)	
C7	0.3353 (2)	0.04845 (6)	0.3848 (3)	0.0230 (4)	
H7	0.3475	0.0204	0.3741	0.028*	
C8	0.4031 (2)	0.07100 (6)	0.5549 (3)	0.0201 (4)	
C9	0.5414 (2)	0.08705 (6)	0.8597 (3)	0.0186 (4)	
C10	0.6438 (2)	0.07586 (6)	1.0502 (3)	0.0191 (4)	
C11	0.6993 (3)	0.03679 (6)	1.0741 (3)	0.0255 (5)	
H11	0.6700	0.0177	0.9659	0.031*	
C12	0.7964 (3)	0.02540 (7)	1.2529 (4)	0.0294 (5)	
H12	0.8349	−0.0012	1.2681	0.035*	
C13	0.8354 (3)	0.05361 (7)	1.4074 (3)	0.0270 (5)	
C14	0.7845 (3)	0.09240 (7)	1.3922 (3)	0.0260 (5)	
H14	0.8147	0.1112	1.5020	0.031*	
C15	0.6878 (2)	0.10357 (6)	1.2123 (3)	0.0228 (4)	
H15	0.6510	0.1303	1.1987	0.027*	
C16	0.6720 (2)	0.18499 (6)	0.7173 (3)	0.0197 (4)	
C17	0.8292 (2)	0.17949 (6)	0.8061 (4)	0.0255 (4)	
H17	0.8473	0.1685	0.9469	0.031*	
C18	0.9588 (3)	0.19039 (7)	0.6845 (4)	0.0329 (5)	
H18	1.0670	0.1865	0.7411	0.039*	
C19	0.9309 (3)	0.20687 (7)	0.4815 (4)	0.0348 (5)	
H19	1.0201	0.2141	0.3987	0.042*	
C20	0.7737 (3)	0.21292 (7)	0.3973 (4)	0.0305 (5)	
H20	0.7559	0.2246	0.2584	0.037*	
C21	0.6421 (3)	0.20191 (6)	0.5153 (3)	0.0242 (4)	
H21	0.5341	0.2059	0.4585	0.029*	
O5W	0.2797 (2)	0.22591 (6)	0.2201 (3)	0.0421 (4)	
H5WA	0.307 (5)	0.2545 (13)	0.220 (6)	0.084 (12)*	
H5WB	0.292 (4)	0.2198 (12)	0.069 (7)	0.083 (12)*	

### Atomic displacement parameters (Å^2^)

**Table:**

	*U*^11^	*U*^22^	*U*^33^	*U*^12^	*U*^13^	*U*^23^
S1	0.0222 (2)	0.0171 (3)	0.0259 (3)	0.00014 (18)	0.00387 (19)	−0.0036 (2)
F1	0.0526 (9)	0.0387 (8)	0.0310 (8)	0.0113 (7)	−0.0197 (7)	−0.0006 (6)
O1	0.0263 (7)	0.0156 (7)	0.0192 (7)	0.0000 (5)	−0.0028 (6)	−0.0017 (5)
O2	0.0371 (9)	0.0290 (9)	0.0277 (8)	0.0027 (7)	−0.0124 (7)	0.0002 (7)
O3	0.0407 (9)	0.0296 (9)	0.0272 (8)	0.0021 (7)	−0.0123 (7)	−0.0066 (7)
O4	0.0244 (7)	0.0239 (8)	0.0367 (9)	0.0046 (6)	0.0012 (6)	0.0001 (7)
C1	0.0187 (9)	0.0165 (9)	0.0193 (9)	−0.0003 (7)	0.0024 (7)	−0.0018 (8)
C2	0.0176 (8)	0.0182 (10)	0.0217 (10)	−0.0013 (7)	0.0021 (7)	−0.0008 (8)
C3	0.0203 (9)	0.0175 (10)	0.0251 (10)	0.0010 (7)	0.0019 (8)	0.0008 (8)
C4	0.0190 (9)	0.0252 (11)	0.0222 (10)	0.0010 (8)	−0.0012 (8)	0.0030 (8)
C5	0.0244 (10)	0.0320 (12)	0.0245 (11)	−0.0010 (9)	−0.0024 (8)	−0.0011 (9)
C6	0.0229 (9)	0.0244 (11)	0.0215 (10)	−0.0031 (8)	−0.0005 (8)	−0.0029 (8)
C7	0.0258 (10)	0.0187 (10)	0.0243 (10)	0.0000 (8)	−0.0008 (8)	−0.0032 (8)
C8	0.0193 (9)	0.0192 (10)	0.0220 (10)	−0.0003 (7)	0.0010 (7)	0.0008 (8)
C9	0.0187 (9)	0.0173 (10)	0.0201 (9)	−0.0017 (7)	0.0022 (7)	−0.0020 (8)
C10	0.0181 (9)	0.0205 (10)	0.0188 (9)	−0.0006 (7)	0.0021 (7)	−0.0001 (8)
C11	0.0313 (11)	0.0204 (11)	0.0243 (11)	0.0020 (8)	−0.0028 (9)	−0.0033 (8)
C12	0.0356 (11)	0.0217 (11)	0.0302 (12)	0.0049 (9)	−0.0043 (9)	0.0009 (9)
C13	0.0275 (10)	0.0314 (12)	0.0215 (10)	0.0036 (9)	−0.0044 (8)	0.0025 (9)
C14	0.0296 (11)	0.0265 (11)	0.0216 (10)	0.0001 (9)	−0.0025 (8)	−0.0049 (9)
C15	0.0265 (10)	0.0191 (10)	0.0228 (10)	0.0024 (8)	0.0016 (8)	−0.0015 (8)
C16	0.0187 (9)	0.0159 (9)	0.0245 (10)	−0.0013 (7)	0.0018 (8)	−0.0034 (8)
C17	0.0235 (10)	0.0229 (11)	0.0298 (11)	−0.0006 (8)	−0.0021 (9)	−0.0006 (9)
C18	0.0188 (9)	0.0330 (13)	0.0467 (14)	−0.0010 (9)	0.0008 (9)	−0.0024 (11)
C19	0.0313 (11)	0.0295 (13)	0.0449 (14)	−0.0048 (10)	0.0140 (10)	−0.0007 (11)
C20	0.0391 (12)	0.0264 (12)	0.0262 (11)	−0.0021 (10)	0.0044 (9)	0.0013 (9)
C21	0.0245 (10)	0.0212 (11)	0.0263 (11)	−0.0003 (8)	−0.0032 (8)	−0.0019 (8)
O5W	0.0574 (12)	0.0293 (10)	0.0406 (11)	−0.0024 (8)	0.0123 (9)	−0.0008 (8)

### Geometric parameters (Å, °)

**Table:**

S1—O4	1.4883 (15)	C10—C11	1.394 (3)
S1—C1	1.768 (2)	C10—C15	1.401 (3)
S1—C16	1.792 (2)	C11—C12	1.385 (3)
F1—C13	1.361 (2)	C11—H11	0.9500
O1—C8	1.377 (2)	C12—C13	1.372 (3)
O1—C9	1.382 (2)	C12—H12	0.9500
O2—C4	1.379 (2)	C13—C14	1.370 (3)
O2—C5	1.424 (3)	C14—C15	1.386 (3)
O3—C6	1.372 (2)	C14—H14	0.9500
O3—C5	1.431 (3)	C15—H15	0.9500
C1—C9	1.362 (3)	C16—C21	1.382 (3)
C1—C2	1.446 (3)	C16—C17	1.394 (3)
C2—C8	1.392 (3)	C17—C18	1.388 (3)
C2—C3	1.412 (3)	C17—H17	0.9500
C3—C4	1.365 (3)	C18—C19	1.380 (4)
C3—H3	0.9500	C18—H18	0.9500
C4—C6	1.396 (3)	C19—C20	1.386 (3)
C5—H5A	0.9900	C19—H19	0.9500
C5—H5B	0.9900	C20—C21	1.389 (3)
C6—C7	1.370 (3)	C20—H20	0.9500
C7—C8	1.390 (3)	C21—H21	0.9500
C7—H7	0.9500	O5W—H5WA	0.99 (4)
C9—C10	1.463 (3)	O5W—H5WB	0.97 (4)
			
O4—S1—C1	105.95 (9)	C11—C10—C9	119.98 (18)
O4—S1—C16	107.47 (9)	C15—C10—C9	121.54 (18)
C1—S1—C16	97.82 (9)	C12—C11—C10	121.2 (2)
C8—O1—C9	107.01 (15)	C12—C11—H11	119.4
C4—O2—C5	105.97 (16)	C10—C11—H11	119.4
C6—O3—C5	105.66 (16)	C13—C12—C11	118.1 (2)
C9—C1—C2	107.74 (17)	C13—C12—H12	121.0
C9—C1—S1	127.35 (15)	C11—C12—H12	121.0
C2—C1—S1	124.90 (15)	F1—C13—C14	118.50 (19)
C8—C2—C3	120.43 (18)	F1—C13—C12	118.2 (2)
C8—C2—C1	104.83 (17)	C14—C13—C12	123.3 (2)
C3—C2—C1	134.74 (19)	C13—C14—C15	118.3 (2)
C4—C3—C2	114.14 (19)	C13—C14—H14	120.9
C4—C3—H3	122.9	C15—C14—H14	120.9
C2—C3—H3	122.9	C14—C15—C10	120.8 (2)
C3—C4—O2	126.70 (19)	C14—C15—H15	119.6
C3—C4—C6	124.11 (19)	C10—C15—H15	119.6
O2—C4—C6	109.16 (17)	C21—C16—C17	121.89 (19)
O2—C5—O3	108.26 (16)	C21—C16—S1	121.13 (15)
O2—C5—H5A	110.0	C17—C16—S1	116.96 (16)
O3—C5—H5A	110.0	C18—C17—C16	118.6 (2)
O2—C5—H5B	110.0	C18—C17—H17	120.7
O3—C5—H5B	110.0	C16—C17—H17	120.7
H5A—C5—H5B	108.4	C19—C18—C17	120.1 (2)
C7—C6—O3	127.0 (2)	C19—C18—H18	119.9
C7—C6—C4	123.18 (19)	C17—C18—H18	119.9
O3—C6—C4	109.81 (18)	C18—C19—C20	120.6 (2)
C6—C7—C8	112.86 (19)	C18—C19—H19	119.7
C6—C7—H7	123.6	C20—C19—H19	119.7
C8—C7—H7	123.6	C19—C20—C21	120.3 (2)
O1—C8—C7	124.23 (18)	C19—C20—H20	119.8
O1—C8—C2	110.50 (17)	C21—C20—H20	119.8
C7—C8—C2	125.26 (19)	C16—C21—C20	118.5 (2)
C1—C9—O1	109.91 (17)	C16—C21—H21	120.8
C1—C9—C10	135.77 (18)	C20—C21—H21	120.8
O1—C9—C10	114.29 (16)	H5WA—O5W—H5WB	100 (3)
C11—C10—C15	118.48 (19)		
			
O4—S1—C1—C9	−152.40 (17)	C2—C1—C9—O1	0.7 (2)
C16—S1—C1—C9	96.85 (18)	S1—C1—C9—O1	−177.99 (13)
O4—S1—C1—C2	29.11 (18)	C2—C1—C9—C10	179.0 (2)
C16—S1—C1—C2	−81.65 (17)	S1—C1—C9—C10	0.3 (3)
C9—C1—C2—C8	−0.6 (2)	C8—O1—C9—C1	−0.5 (2)
S1—C1—C2—C8	178.14 (14)	C8—O1—C9—C10	−179.20 (15)
C9—C1—C2—C3	179.6 (2)	C1—C9—C10—C11	−172.4 (2)
S1—C1—C2—C3	−1.7 (3)	O1—C9—C10—C11	5.7 (3)
C8—C2—C3—C4	−0.4 (3)	C1—C9—C10—C15	7.8 (3)
C1—C2—C3—C4	179.5 (2)	O1—C9—C10—C15	−173.98 (17)
C2—C3—C4—O2	−178.00 (18)	C15—C10—C11—C12	−0.3 (3)
C2—C3—C4—C6	−0.4 (3)	C9—C10—C11—C12	180.00 (19)
C5—O2—C4—C3	−176.2 (2)	C10—C11—C12—C13	0.6 (3)
C5—O2—C4—C6	5.9 (2)	C11—C12—C13—F1	178.6 (2)
C4—O2—C5—O3	−10.2 (2)	C11—C12—C13—C14	−0.7 (4)
C6—O3—C5—O2	10.6 (2)	F1—C13—C14—C15	−178.81 (19)
C5—O3—C6—C7	174.8 (2)	C12—C13—C14—C15	0.5 (3)
C5—O3—C6—C4	−7.0 (2)	C13—C14—C15—C10	−0.1 (3)
C3—C4—C6—C7	1.0 (3)	C11—C10—C15—C14	0.0 (3)
O2—C4—C6—C7	178.98 (19)	C9—C10—C15—C14	179.77 (18)
C3—C4—C6—O3	−177.25 (18)	O4—S1—C16—C21	−22.09 (19)
O2—C4—C6—O3	0.7 (2)	C1—S1—C16—C21	87.42 (18)
O3—C6—C7—C8	177.22 (19)	O4—S1—C16—C17	156.30 (16)
C4—C6—C7—C8	−0.7 (3)	C1—S1—C16—C17	−94.18 (17)
C9—O1—C8—C7	179.68 (18)	C21—C16—C17—C18	−1.8 (3)
C9—O1—C8—C2	0.1 (2)	S1—C16—C17—C18	179.81 (17)
C6—C7—C8—O1	−179.52 (17)	C16—C17—C18—C19	1.0 (3)
C6—C7—C8—C2	0.0 (3)	C17—C18—C19—C20	0.4 (4)
C3—C2—C8—O1	−179.85 (16)	C18—C19—C20—C21	−0.9 (4)
C1—C2—C8—O1	0.3 (2)	C17—C16—C21—C20	1.2 (3)
C3—C2—C8—C7	0.6 (3)	S1—C16—C21—C20	179.56 (16)
C1—C2—C8—C7	−179.26 (18)	C19—C20—C21—C16	0.2 (3)

### Hydrogen-bond geometry (Å, °)

**Table:**

*D*—H···*A*	*D*—H	H···*A*	*D*···*A*	*D*—H···*A*
C18—H18···O4^i^	0.95	2.46	3.375 (3)	162.
C19—H19···O5w^i^	0.95	2.49	3.433 (3)	171.
O5W—H5WA···O4^ii^	0.99 (4)	1.87 (4)	2.834 (2)	164 (3)
O5W—H5WB···O4^iii^	0.97 (4)	1.98 (4)	2.908 (2)	161 (3)

Symmetry codes: (i) *x*+1, *y*, *z*; (ii) *x*, −*y*+1/2, *z*−1/2; (iii) *x*, *y*, *z*−1.
